# The role of gender in the prevalence of eating disorders

**DOI:** 10.1192/j.eurpsy.2024.1163

**Published:** 2024-08-27

**Authors:** G. Strada Herrera, I. López Claramunt, B. Serván Rendón-Luna, L. M. Sanz Martín

**Affiliations:** Instituto de Psiquiatría y Salud Mental, Hospital Clínico San Carlos, Madrid, Spain

## Abstract

**Introduction:**

Eating disorders have a key paper at the ongoing society. A key symptom of the Anorexia Nervosa and the Bulimia Nervosa is the alteration of the corporal image which observes that it continues being present after remitting the most flowery symptomatology. In terms of gender, we can observe that the esting disorders have a higher incidence in the feminine gender.

**Objectives:**

Research how body image affects eating disorders and how the role of gender is a risk factor for developing Anorexia Nervosa or Bulimia Nervosa.

**Methods:**

A systematic review was conducted using PubMed. Twelve studies were identified in order to do this review.

**Results:**

At the twelve surveys included at the review we can observe that the incidence of Anorexia Nervosa and Bulimia Nervosa is higher in women than men. There are many facts that take part on the development of eating disorders, but there is consensus to understand them with a biopsicosocial point of view (interaction between the environment and biological facts). Body image disturbance takes part in both men and women, but it affects them in different ways.

**Conclusions:**

Body image disturbances are a crucial factor when considering eating disorders’ symptomatology. One of the main components that affects its alteration is the internalization of standards of beauty. Women tend to focus on thin body types, meanwhile men’s attention tends to point to muscular and defined body types. Nevertheless, it must be taken into account that today’s gender conception may appear as one of the most important roles to understand Anorexia and Bulimia aetiology. Regarding gender, in nowadays society exists a dichotomy where masculinity and femininity lie in total opposites poles; but if the gender approach socially changed, Anorexia and Bulimia might take a different portrayal.

**Disclosure of Interest:**

None Declared

